# Identification of Novel Proteins Interacting with Proprotein Convertase Subtilisin/Kexin 9

**DOI:** 10.31531/2581-4745.1000123

**Published:** 2020-02-27

**Authors:** Quantil M. Melendez, Catherine J. Wooten, Sreevidhya T. Krishnaji, Kevin Knagge, David Kirchner, Dayami Lopez

**Affiliations:** 1 Department of Pharmaceutical Sciences, Biomanufacturing Research Institute and Technology Enterprise (BRITE), College of Arts and Sciences, North Carolina Central University, Durham, USA; 2 Indian Institute of Science Education and Research Bhopal, Madhya Pradesh, India; 3 David H Murdock Research Institute, Kannapolis, USA

**Keywords:** Hypercholesterolemia, PCSK9, Protein-protein interactions, Endogenous regulator, A1AT, APOH, AMBP

## Abstract

High levels of cholesterol, especially as low-density lipoprotein (LDL), are a well-known risk factor for atherosclerotic-related diseases. The key atherogenic property of LDL is its ability to form atherosclerotic plaque. Proprotein convertase subtilisin/kexin-9 (PCSK9) is an indirect regulator of plasma LDL levels by controlling the number of LDL receptor molecules expressed at the plasma membrane, especially in the liver. Herein, we performed a combination of affinity chromatography, mass spectrometry analysis and identification, and gene expression studies to identify proteins that interact with PCSK9. Through these studies, we identified three proteins, alpha-1-antitrypsin (A1AT), alpha-1-microglobulin/bikunin precursor (AMBP), and apolipoprotein H (APOH) expressed by C3A cells that interact with PCSK9. The expression levels of A1AT and APOH increased in cells treated with MITO+ medium, a condition previously shown to affect the function of PCSK9, as compared to treating with Regular (control) medium. However, AMBP expression did not change in response to the treatments. Additional studies are required to determine which of these proteins can modulate the expression/function of PCSK9. The identification of endogenous modulators of PCSK9’s function could lead to the development of novel diagnostic tests or treatment options for patients suffering hypercholesterolemia in combination with other chronic metabolic diseases.

## Introduction

Hypercholesterolemia, the primary cause of atherosclerotic-related diseases, is still considered a severe health problem worldwide [1,2]. The major determinant of plasma low density lipoprotein (LDL) levels is the hepatic LDL receptor [1,2]. Proprotein convertase subtilisin/kexin-9 (PCSK9) is a well-known indirect regulator of the amount of LDL in the bloodstream since this convertase controls the plasma membrane expression of the LDL receptor [3–5]. After its secretion into the serum, the PCSK9’s C-terminal domain interacts with the LDL receptor’s epidermal growth factor-like repeat A (EGF-A) at the surface of cells [5–7]. Then, the PCSK9/LDL receptor complex enters the endosomal pathway [6,7]. Unlike the interaction between a lipoprotein particle and the LDL receptor, the affinity of PCSK9 for the receptor increases as a result of the acidic pH of the endosome [7,8]. Accordingly, the PCSK9/LDL receptor complex is sent to the lysosome to be degraded [7,8].

In humans, treatment with atorvastatin induces PCSK9 protein levels and the function of the LDL receptor simultaneously, an effect accentuated by increasing the dose of atorvastatin [9]. Interestingly, as more PCSK9 protein is produced due to a higher dose of atorvastatin, the extent of the atorvastatin-dependent reduction in LDL-cholesterol levels is diminished [9]. Similar results have been seen for other statin compounds [10–14]. The observed effects of statins on PCSK9, and the discovery of the connection between loss-of-function (LOF) mutations of PCSK9, hypocholesterolemia, and a decreased risk of developing cardiovascular diseases (CVD) [15,16], justified the manufacturing of PCSK9 inhibitors for the treatment of hypercholesterolemia [17]. Interestingly, PCSK9 is one of the genes associated with resistance to statins [18]. Currently, two PCSK9 inhibitors, RepathaTM [19] and Praluent® [20], are approved for their clinical use to prevent degradation of the LDL receptor by PCSK9 and reducing hypercholesterolemia.

We have previously reported that the availability of elevated levels of PCSK9 protein to bind the LDL receptor is not sufficient to determine the number of PCSK9/LDL receptor protein complexes that form in a cell [21]. Exposing hepatic cells to a medium supplemented with BDTM MITO+ serum extender (MITO+ medium) results in statistically significant lower levels of PCSK9/LDL receptor complexes despite having elevated levels of PCSK9 protein, both secreted and intracellularly, as compared to cells exposed to standard (10% fetal bovine serum or FBS) or delipidated medium [21]. We also discovered that the majority of the PCSK9 molecules produced as a result of incubating the cells with the MITO+ medium was inhibited by a secreted factor [21]. However, neither LDL or annexin A2, the two factors associated with decreased interaction of PCSK9 with the LDL receptor [22–24], were responsible for preventing complex formation between PCSK9 and the receptor upon treatment with the MITO+ medium [21].

In the current study, we sat out to identify proteins secreted by the hepatic cells that directly interacted with PCSK9. Three proteins secreted by hepatic cells that interact with PCSK9 were identified in this study. If any of these proteins results to be the endogenous inhibitor of PCSK9’s function, its identification could help in the design of more efficient drugs/biologicals to treat hypercholesterolemia.

## Materials and Methods

### Materials

American Type Culture Collection (Manassas, VA) was the source of the human hepatocyte-like C3A cell line. Low glucose (LG; 5.55 mM) Dulbecco’s modified Eagle’s medium (D-MEM), penicillin/streptomycin solution, gentamycin sulfate, phosphate buffered saline (PBS), standard FBS, precast NuPAGE™ 4–12% Bis-Tris protein gels, and the NuPAGE® MES SDS Running (20X), Transfer (20X) and LDS Sample (4X) buffers, Ponceau S stain, the AB reverse transcriptase system, and AB SYBR Green PCR Master Mix were from Invitrogen ThermoFisher Scientific (Carlsbad, CA). BDTM MITO+ serum extender (MITO+) was purchased from BD Biosciences (Sparks, MD). Slide-A-LyzerTM Dialysis Cassettes (2K MWCO, 30 mL), the BCA protein assay, SuperSignal West Pico chemiluminescent substrate, protease and phosphatase inhibitors, dimethyl 3,3′-dithiobispropionimidate (DTBP), disuccinimidyl glutarate (DSG), streptavidin magnetic beads, and the ELISA tetramethylbenzidine (TMB) substrate were obtained from Pierce Thermo Scientific (Rockford, IL). The CircuLex wild-type (WT) PCSK9 protein, the avidin-horseradish peroxidase (HRP) for Western blotting analysis, the HisPur Ni-NTA resin, 3 kDa centrifuge concentrators, and other chemicals not mentioned in this section were from Fisher Scientific (Pittsburgh, PA).

The mouse anti-PCSK9 antibody was purchased from Cayman Chemicals (Ann Arbor, MI). The actin-specific antibody HRP-labeled secondary antibodies, and BSA (ELISA grade) were from Santa Cruz Biotechnology (Santa Cruz, CA). The annexin A2 antibody was obtained from Aviva Systems Biology (San Diego, CA). The rat anti-human PCSK9 antibody for coating, the human PCSK9/LDLR Complex Assay, recombinant (r) PCSK9 carrier-free (CF) protein, the biotinylated sheep anti-PCSK9 antibody (used for Western blotting analysis, immunoprecipitation, and detection for ELISA), the alpha-1-antitrypsin (A1AT; MAB1268), the apolipoprotein H (APOH; AF5087), the streptavidin-HRP for ELISA, and the rLDLR CF protein came from R&D Systems (Minneapolis, MN). The alpha-1-microglobulin/bikunin precursor (AMBP) ELISA kit was from Abnova (Walnut, CA). The Molecular Research Center (Cincinnati, OH) was the source of TRI Reagent. The DNA removal kit was from Promega (Madison, WI). Primers for real-time PCR were synthesized by Eurofins Genomics (Huntsville, AL).

### C3A Cell Culture

Human hepatocyte-like C3A cells were maintained in D-MEM supplemented with 10% FBS and antibiotics (Regular medium) as previously described [21]. Secretion of the putative endogenous inhibitor of PCSK9 was induced by setting the cells in T-75 flasks at the density of 2 × 10^6^ cells per flask in the Regular medium. Twenty-four hours later, the medium was replaced with MITO+ medium and incubated for 72 hours as previously described [21]. The conditioned medium collected at 72 hours was saved at −80 °C. The processing was repeated until sufficient conditioned medium was collected for follow-up processing. For some experiments, cells were plated in 12-well plates and were treated with Regular (control) and MITO+ media as previously described [21]. Again, the conditioned medium was collected and concentrated for analysis by Western blotting. Cells were employed in the preparation of RIPA proteins, non-denaturing proteins, or RNA samples as described below.

### Preparation of Concentrated Medium Samples

Equivalent volumes of conditioned medium were mixed with equal volumes of saturated ammonium sulfate (259.55 g/500 mL) for a final saturation of 50%. This methodology to precipitate medium proteins has been used extensively [25]. The mixtures were incubated at 4 oC with shaking overnight. Samples were centrifuged at 5,710 × g for 20 minutes at 4 °C. After disposing of the supernatant, the pellets were resuspended in ice-cold PBS supplemented with protease and phosphate inhibitors. The volume used to resuspend the pellets was 1/10^th^ of the original volume of conditioned medium. The salt was removed by dialyzing in PBS with protease/phosphatase inhibitors at 4 °C, for 4 hours first and then overnight, using Slide-A-LyzerTM Dialysis Cassettes. Protein concentrations were determined using a BCA protein assay kit. Negative control samples were prepared by precipitating and dialyzing MITO+ medium that was not exposed to cells (non-conditioned MITO+ medium).

### RIPA Protein Preparation

Lysates were prepared using cold RIPA buffer supplemented with protease and phosphate inhibitors as previously described [21]. Cellular lysates (supernatants) were stored at −80°C until needed for the next step. Protein concentrations were determined with the BCA protein assay.

### Protein Electrophoresis

Equivalent amounts of protein samples (concentrated medium proteins, CircuLex WT PCSK9 protein, or RIPA proteins, depending on the experiment) were denatured in NuPAGE® LDS Sample buffer supplemented with 20 mM DTT at 70 °C for 5 minutes and subjected to electrophoresis on precast NuPAGE™ 4–12% Bis-Tris protein gels and NuPAGE® MES SDS Running Buffer. Electroblotting onto nitrocellulose membranes using NuPAGE® Transfer Buffer and staining with 0.1% Ponceau S (in 5% acetic acid) to verify equal protein loading were performed using standard methods. Gels for Far-Western blotting analysis were run with samples prepared in a nondenaturing/nonreducing sample buffer (NRNDSB: 140 mM Tris-HCl pH 6.8, 10% glycerol, 0.002% BPB; without heating samples) or under denatured/reduced conditions (RDSB: 140 mM Tris-HCl pH 6.8, 10% SDS, 10% glycerol, 0.002% BPB, and 20 mM DTT; samples were heated as usual). Electrophoresis was performed in 10% native PAGEs using TRIS-Glycine running buffer without SDS or in NuPAGE™ 4–12% Bis-Tris protein gels as described for samples prepared using the LDS sample buffer.

### Standard Western Blotting Analysis

This technique was performed essentially as previously described [21]. Blocking with 2% BSA-TBS or 5% non-fat dry milk-TTBS (depending on whether the antibody was biotinylated or not, respectively) was performed for 30 minutes at room temperature. Primary antibodies used were mouse anti-PCSK9 (Cayman; diluted 1:1000), rabbit anti-annexin A2 (diluted 1:250), mouse anti-A1AT (diluted 1 μg/mL), goat anti-APOH (diluted 1 μg/mL), goat anti-actin (used as internal control in some studies; diluted 1:250), and biotinylated sheep anti-PCSK9 antibody (R&D Systems; diluted 1:150). After incubating with HRP-labeled secondary antibodies (diluted 1:2,000) or avidin-HRP (diluted 1:500), depending on the primary antibody, immunoreactive proteins were detected using the SuperSignal West Pico Chemiluminescence Substrate. Several exposures ranging from 0.5 s to 30 minutes were made using a Kodak Image Station 4000R Pro Imaging System (Bend, OR) and the Carestream Molecular Imaging Software-Standard Edition-v.5.4.2.18893 (New Haven, CT). When needed, quantitation of the Western blot signals was performed using the Imaging software.

### Far-Western Blotting Analysis

This procedure was carried out as a variation of the Standard Western blotting assay. After blocking with 2% BSA-TBS or 5% non-fat dry milk-TTBS (depending on whether the antibody was biotinylated or not, respectively) for 1 hour at room temperature, the membranes were incubated with 0.1 μg/mL of rPCSK9 (R&D Systems) in 2% BSA/TBS overnight at 4 °C with shaking. In the case of the far-Western blotting for annexin A2, the membrane containing control and CircuLex WT PCSK9 samples was incubated with 100 μg/mL concentrated medium proteins in 2% BSA/TBS. After washing five times with TTBS and two times with TBS, the membrane was incubated with either the biotinylated PCSK9 antibody (dilution 1:150) or the annexin A2 specific antibody (dilution 1:250) for 2 hours at room temperature. The antibodies were then detected by incubating with avidin-HRP (diluted 1:500) or anti-rabbit-HRP (diluted 1:2000) in 2% BSA/TBS for 30 minutes at room temperatures. As for standard Western blotting analysis, washes were performed after each incubation step with an antibody or avidin-HRP. Again, the signal was detected using the West Pico Chemiluminescence Substrate. A control membrane was incubated with the PCSK9 antibody after incubating overnight in blocking solution. Exposures were performed as for standard Western blotting analysis.

### Crosslinking Studies

For this experiment, 1.2 mg of concentrated serum proteins were mixed with 1 mM of either DTBP or DSG. A mixture of DMSO/PBS was used as the vehicle for the control samples. In another experiment, increasing amounts of DSG (0, 1, and 2 mM) and medium proteins were used. The mixtures of protein/crosslinker were incubated for 1 hour at room temperature with shaking. The reactions were stopped by adding 1M Tris-HCl, pH 7.0 followed by incubation for 5 minutes at room temperature. Aliquots of the crosslinked samples were prepared with NuPAGE® LDS Sample buffer without DTT or heating. Electrophoresis was performed under non-reducing conditions. Standard Western blotting analysis was used to detect protein complexes involving PCSK9. The remaining cross-linked samples were used in immunoprecipitation reactions using the biotinylated sheep anti-PCSK9 antibody. Briefly, the crosslinked sample was mixed with the PCSK9 antibody (final concentration = 0.25 μg/mL) in a total volume of 1.5 mL. The mixtures were incubated overnight at 4 °C with shaking. Fifty μL of PBS equilibrated streptavidin magnetic beads were added to each reaction followed by incubating for 1 hour at room temperature. The immunoprecipitated (IP) complexes/magnetic beads were separated and washed using a magnetic stand. Elution of the IP complexes was performed by resuspending in 2M glycine pH 2.0. After incubation at room temperature for 10 minutes, the magnetic stand was used once again to separate the magnetic beads from the eluant containing the IP cross-linked samples. The glycine solution was neutralized by adding 1M Tris-HCl pH 8.0 (1/10^th^ of the original glycine volume) to the samples. Electrophoresis under non-reduced conditions and standard Western blotting analysis were performed as described for the cross-linked samples before immunoprecipitation.

### PCSK9 Affinity Chromatography

The entire process was carried out at 4 °C. For this, chromatography columns were filled with equilibrated HisPur Ni-NTA resin. After washing with cold equilibration buffer (20 mM sodium phosphate, 300 mM sodium chloride, 10 mM imidazole pH 7.4), 60 mg of rPCSK9 (His-tagged) diluted in 1 mL of equilibration buffer was added to corresponding columns and permitted to bind. The columns were washed with equilibration buffer, and the flow-through was collected. The flow-through was then allowed to run through each column for a total of three times before it was discarded. An aliquot was saved for further analysis (FT after rPCSK9). After washing the columns with equilibration buffer one more time (FT after EB wash), 2 mL of concentrated medium proteins was added to each column, and the flow-through was saved. Once again, the flow-through was allowed to run through each column for a total of three times before an aliquot was saved for further analysis (FT after MP). The columns were washed with cold wash buffer (20 mM sodium phosphate, 300 mM sodium chloride, 25 mM imidazole pH 7.4). The flow-through was discarded after saving an aliquot for analysis (FT after WB wash). Proteins interacting with PCSK9 were eluted with 25 mL of elution buffer (20 mM sodium phosphate, 300 mM sodium chloride, 250 mM imidazole pH 7.4). One mL fractions were collected under the columns. Once 25 aliquots were collected, the next 10 mL of elution buffer was also collected in a single tube and an aliquot saved for further analysis (FT after additional EB). Protein levels in each fraction were determined using the BCA assay. Electrophoresis and standard Western blotting analysis using the mouse PCSK9 antibody were done as described above. In some experiments, the medium samples were prepared at different pHs (7.4 and 5.0) before adding them to the columns. Aliquots containing PCSK9 were pooled together and concentrated using 3 kDa concentrators.

### High-Resolution LC-MS/MS Proteomic Analysis

The two sample aliquots were sent out to the David H. Murdock Research Institute (DHMRI), Kannapolis, NC, for MS based proteomic analysis - to identify the proteins interacting with PCSK9 in the affinity columns.

### Proteomics Sample Preparation

Protein concentration of the two samples was determined using micro BCA assay (Thermo) following the manufacturer’s protocol for use with microplates. The concentrations were: sample 1 (pH 7.4) −14.98 μg/mL and sample 3 (pH 5.5) - 4.70 μg/mL. These concentrations were too low to carry out the protein enzyme digest. Therefore, the samples were again concentrated using 3kD centrifugal MWCO filters (Millipore) until the sample volume was ~20 μL. The samples were recovered into new micro-tubes and then placed in a vacufuge (Eppendorf) and dried down. The proteins were reconstituted in 20 μL of 50 mM ammonium bicarbonate. Then 2 μL of 100 mM dithiothreitol was added to each of the tubes, and the samples were incubated at 80°C for 30 min. After reduction, the samples were alkylated by the addition of 2 μL of 200 mM iodoacetamide to the samples and incubated at room temperature in the dark for 20 min. Then, 2 μL of 100 ng/μL Trypsin Gold® (Promega) was added, and the tubes were incubated at 37°C for 5 hours and then 4°C overnight. The reactions were then quenched by adding 0.5 μL of 5% acetic acid to the reaction tubes. Then the samples were vacuufuged for 30 min at 30° C and dried down. The two samples were then reconstituted in 25 μL LC loading buffer (2% acetonitrile, 0.1% formic acid), and briefly sonicated to solubilize the peptides, and all 25 μL of was transferred into clean, labeled LC/MS vials for data collection and analysis.

### Capillary Chromatography and LC-MS/MS Analysis

Fifty femto moles of standard Yeast Enolase tryptic digest was injected and analyzed as a quality control sample in the beginning and at the end of data collection. Ten μL of each sample was injected for analysis on an LTQ-Orbitrap XL MS. An online reversed phase C18 (Zorbax, Agilent) sample trapping, cleanup and focusing was done for the first 10 min of each analysis. Then a 33 min elution gradient was used for analytical C18 (PepMap, Thermo) separation of the tryptic peptides. The MS method employed was Full scan profile MS at 60,000 resolution (350 – 1800 m/z) followed by top 3 in abundance selection for centroided tandem CID MSMS and a decision tree-based ETD activation fragmentation option.

### Database Searching and PEAKS Analysis

Data analysis was done by importing the RAW data files into PEAKS Studio 7.5 (BSI Software). PEAKS database search engine query parameters were set as: Parent mass error tolerance was 50.0 ppm and the fragment mass error tolerance was 0.5 Da. Precursor mass type was set for monoisotopic masses and trypsin enzyme cleavage rules. Maximum missed enzymatic cleavages was 3, and any non-specified cleavages can be at either end of a peptide. A fixed Carbamidomethylation (57.02 Da) of cysteine modification was needed to reflect the reduction and alkylation of the proteins, and a variable oxidation (15.99 Da) of methionine modification was used. In conjunction with the two aforementioned peptide modifications used in the PEAKS DB search, an additional search called PEAKS PTM search was also utilized to search the database with 483 variable “common” protein modifications and amino acid mutations or conversions. This was done to help identify more peptides that may have modifications, but the measured parent mass does not match to any predicted peptide mass in silico during a standard database search. The database used in the query was Uniprot-SwissProt with the taxon set to human sequences. The False Discovery Rate (FDR) estimation used was 0.0 and a peptide hit threshold of 30.0 (−10logP).

### RNA Preparation and Quantitative Real-time Polymerase Chain Reaction (qRT-PCR)

Total RNA was prepared using the acid guanidinium thiocyanate-phenol-chloroform extraction method employing TRI Reagent [26]. Concentration and purity of the RNA samples were determined using a Nanodrop 2000. The integrity of the RNA was also confirmed with RNA electrophoresis. DNase I treatment and reverse transcriptase reactions were carried out using standard methods. qRT-PCR reactions were performed using 100 ng of ssDNA, the Applied Biosystems SYBR Green PCR Master Mix, and the AB real-time PCR system, with the following parameters: denaturation at 95 °C for 10 minutes, followed by 45 cycles of denaturation at 95 °C for 30 seconds, annealing at different temperatures (see below) for 15 seconds, extension at 72 °C for 30 seconds, and photo documentation at 80 °C for 15 seconds. A melt curve was proceeded to determine if each primer set was amplifying as a single band. Primers used in this experiment were: 5’-AGCCTGGTGGAGGTGTATCT-3’ and 5’-GCCATGACTGTCACACTTGC-3’ for PCSK9; 5’GTACCCTCAACCAGCCAGAC-3’ and 5’-GTGTCCCCGAAGTTGACAGT-3’ for A1AT; 5’TGCAGAGAGTACTGCGGTGT-3’ and 5’-TTTATTTGGACCCAGGTTGC-3’ for AMBP; 5’-AACAGAGAGCCAGGACCAAA-3’ and 5’-GCCTGCAGTATGCACAGGTA-3’ (initial set) for SEPP; 5’-CCCTCCACCATCCATACCTA-3’ and 5’CCATGTGTCGTGCAGGTAAT-3’ for APOH, and 5’-GGGACAAGTGGCGTTCAG-3’ and 5’-CGCTGAGCCAGTCAGTGTAG-3’ for 18s rRNA. The annealing temperature for the PCSK9 and 18s rRNA primers was 63 °C, whereas, for the A1AT, AMBP, and APOH, the annealing temperature was 65 °C. Different annealing temperatures were tested for SEPP. Quantitation of the result was done using the comparative CT method as previously described [26].

### Detection of Secreted PCSK9 Protein Levels using ELISA

To detect secreted PCSK9 protein levels, a sandwich ELISA was performed as per a previously published assay [21]. Briefly, a rat anti-human PCSK9 antibody (2 μg/ml) was used to coat the plates overnight. Blocking was carried out with 1% BSA/PBS for 1.5 hours at room temperature. Standards and fractions collected from the affinity chromatography were added to the plate and incubated for 2 hours at room temperature. Detection was carried out using the biotinylated sheep anti-PCSK9 antibody (400 ng/ml) for 2 hours at room temperature. Streptavidin-HRP (diluted 1:200) was then added and incubated for 20 minutes at room temperature. All the washes were done with PBS/0.05% Tween 20 for a total of three times. Substrate solution containing 0.01% H_2_O_2_ and 0.2 g/L TMB was added to the wells and incubated in the dark for 20 minutes at room temperature. The reactions were stopped with 2N sulfuric acid, and the optical density of the yellow colored product was determined using an M5 Spectramax plate reader. Correction for optical imperfections in the plate was carried out by reading the plate at 540 nm, and these readings were subtracted from the 450 nm readings.

### Preparation of Cellular PCSK9/LDL Receptor Complexes for ELISA

This was performed as previously described [21]. Briefly, cells treated as indicated above were first washed twice with PBS, and then incubated rotating at 4 °C with ice-cold, non-denaturing cell lysing buffer (20 mM Tris–HCl, pH 7.5, 150 mM NaCl, 1 mM Na2EDTA, 1 mM EGTA, 1% Triton X-100, and 2.5 mM sodium pyrophosphate) for 30 min. Cell lysates were centrifuged for 10 minutes at 15,000 × g at 4 °C. The supernatants corresponding to non-denatured cell lysates were saved at −80 °C, and their protein concentrations were determined using the BCA protein assay.

### Detection of PCSK9/LDL Receptor Protein Complexes using ELISA

To detect PCSK9/LDL receptor protein complexes in non-denatured cell lysates, sandwich ELISA using the Human PCSK9/LDLR Complex DuoSet ELISA Development System was performed exactly as previously described [21]. Determination of the optical density of the yellow colored product was conducted using an M5 Spectramax plate reader. Correction for optical imperfections in the plate was done by reading the plate at 540 nm and then subtracting these readings from the 450 nm readings.

### Detection of AMBP Protein using ELISA

Medium samples collected from control (Regular medium) and MITO+ medium treated cells were analyzed by AMBP ELISA (Abnova; KA1406) using the manufacturer’s instructions. Briefly, standards (8 total; ranging concentrations 0 – 40 ng/mL) and samples (diluted 1:100) were added to the plate pre-coated with an antibody specific for AMBP. Incubation was then carried out for 2 hours at room temperature. After washing five times as indicated in the protocol, detection was performed using a biotinylated antibody against AMBP supplied with the kit. Incubation with this antibody was carried out for 1 hour at room temperature. After washing, streptavidin-HRP was added to the wells followed by incubation 30 minutes at room temperature. Again, washing was repeated. The substrate solution was then added to the wells according to the protocol, and the plate was incubated for 7 minutes at room temperature. As for the ELISA assays described above, the reactions were stopped, and the resulting color was measured by reading the plate at 450 (signal) and 540 (background correction) nm. Calculations were performed as described above.

### Statistical Analysis

Data from the individual parameters for at least three independent measurements (n=3) were compared employing Student t-test and calculated with the GraphPad Prism 7 software (GraphPad Software, Inc., La Jolla, CA). The significance level was set at α = 0.05.

## Results

We started by collecting large amounts of conditioned MITO medium from C3A cells. The proteins in the medium were precipitated using 50% ammonium sulfate. The pellet collected after centrifuging the precipitated proteins was resuspended in minimum volumes (1/10^th^ of the original volume) of PBS supplemented with protease/phosphatase inhibitors. The ammonium sulfate was removed from concentrated samples by dialysis against PBS supplemented with protease/phosphatase inhibitors.

We then performed Far-Western blotting analysis. For this, aliquots of concentrated medium samples were prepared using non-denatured/nonreduced (NRNDSB) and denatured/reduced (RDSB) sample buffers. The samples were electrophoresed under native and denaturing PAGE conditions as described in Materials and Methods. Membranes obtained after electroblotting were blocked with 2% BSA/TBS for 1 hour and then incubated with 0.1 μg/mL rPCSK9 in 2% BSA/TBS overnight at 4 °C. Incubation with the biotinylated anti-PCSK9 antibody and avidin-HRP was performed as described above. Immunoreactive proteins were detected using the West Pico Chemiluminescence Substrate and imaged as described above.

**Figure 1:**
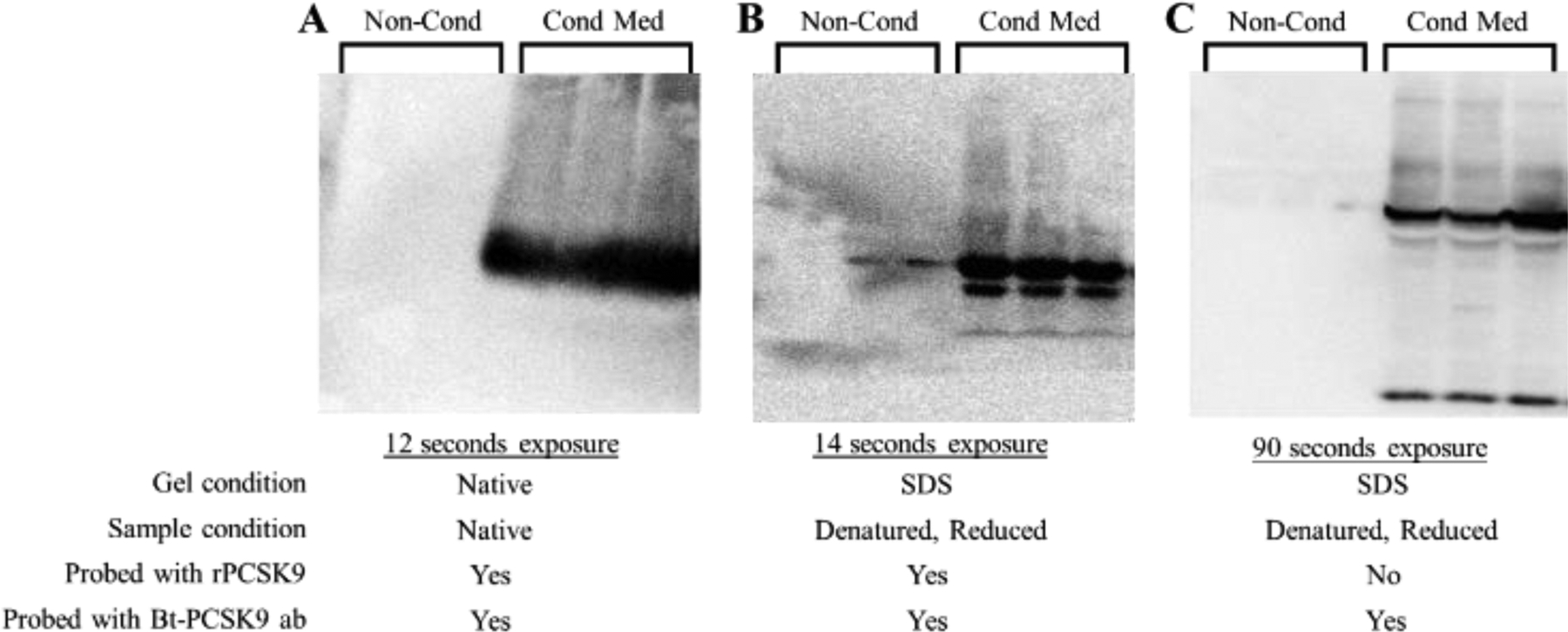
Far-Western blotting studies using recombinant (r) PCSK9 to detect concentrated conditioned medium proteins that interact with PCSK9. Concentrated medium samples (Cond Med) were prepared using ammonium sulfate precipitation followed by desalting using dialysis. Conditions for preparing the samples, electrophoresis, and far-Western blotting analysis using a biotinylated (Bt) PCSK9 specific antibody have been described in Materials and Methods. Typical far-Western blots for non-denaturing/non-reducing (A) and denaturing/reducing (B and C) conditions are shown. “Non-Cond” refers to samples prepared from an MITO+ medium that was not exposed to cells. The exposure time for each blot has been indicated.

Figure 1 shows that separating the samples in non-denatured/nonreduced (1A) and denatured/reduced conditions (1B) had no effect on the number of protein bands detected by Far-Western blotting. The gels shown in Figure 1B and C were run simultaneously in the same electrophoresis system with an equal amount of protein per well. Figure 1C shows the results of a control membrane that was not incubated with rPCSK9 before detection with the biotinylated anti-PCSK9 antibody. When comparing both gels, it was seen that the band identified by the far-Western blotting analysis ran faster (near the 42 kDa protein marker; Fisher BP3603–1) than the band detected in the standard Western blotting (between the 55 and 72 kDa protein marker; about 60 kDa; corresponding to mature PCSK9) (Figure 1). Protein signals in lanes containing samples prepared from non-conditioned MITO+ medium was mainly undetected.

**Figure 2:**
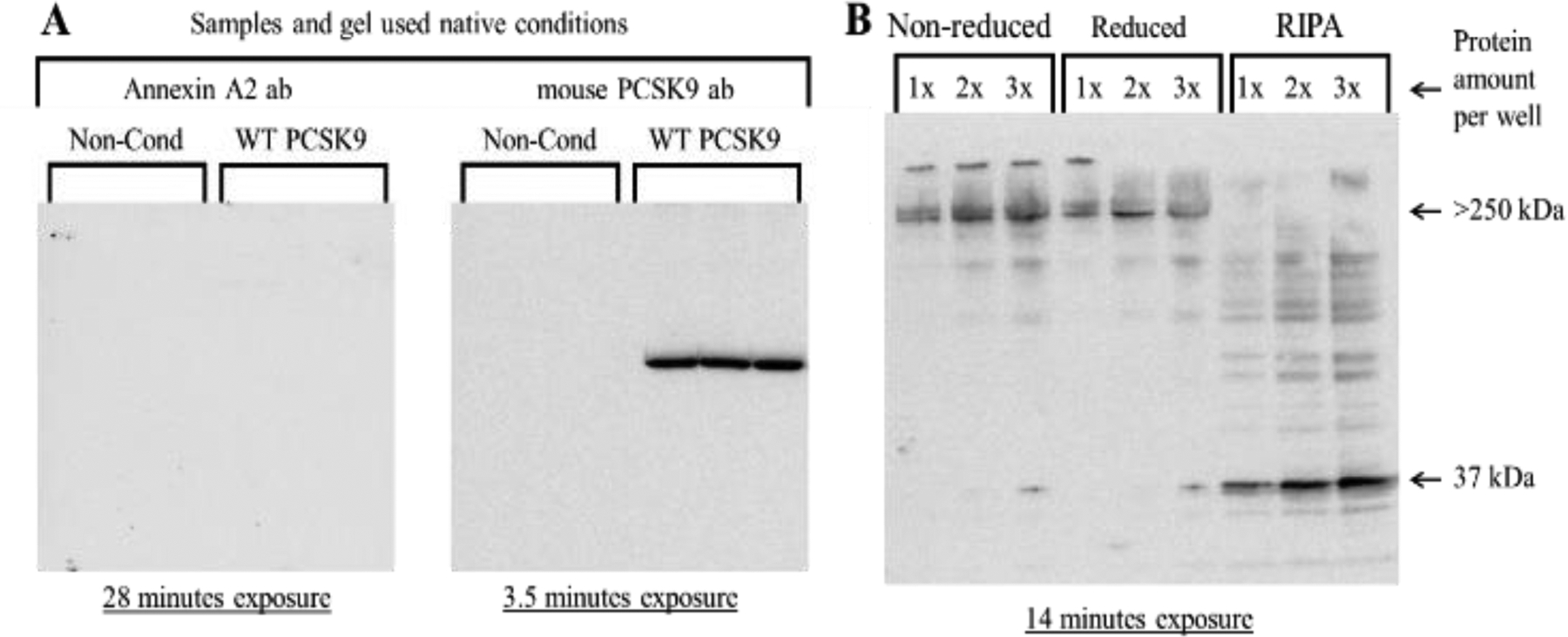
Far-Western blotting studies using concentrated conditioned medium proteins to determine the presence of annexin A2 in these samples. (A) Conditions for preparing the control and WT PCSK9 (CircuLex) samples, electrophoresis using native conditions, and far-Western blotting analysis was described in Materials and Methods. A representative far-Western blot for annexin A2 (left blot) and a standard Western blot for follow-up probing of the same gel with a mouse anti-PCSK9 antibody (right blot) are shown. “Non-Cond” refers to samples prepared from an MITO+ medium that was not exposed to cells. (B) Standard Western blotting analysis for annexin A2 in medium and RIPA samples. Major protein bands have been indicated with arrows. The exposure time for each blot has been indicated.

Since annexin A2 has been identified as a protein that interacts with PCSK9 [23,24], far-Western blotting analysis for annexin A2 was also performed. For this experiment, non-conditioned MITO+ medium and CircuLex WT PCSK9 protein samples were run under native conditions, and the resulting membrane was blocked and then incubated with 100 μg/mL concentrated medium proteins diluted in 2% BSA/TBS overnight. Probing with an annexin A2-specific antibody was done to determine if annexin A2 was bound to the PCSK9 on the membrane. As shown in Figure 2A (left blot), no annexin A2 was detected using this technique. To confirm the presence of PCSK9, the same membrane was probed with a mouse anti-PCSK9 antibody. As shown, PCSK9 was detected in the samples containing WT PCSK9 (Figure 2A; right blot). Increasing amounts of the concentrated conditioned medium samples were also analyzed for annexin A2 levels using standard Western blotting analysis. Increasing aliquots from RIPA proteins were also analyzed as control samples. As shown in Figure 2B, annexin A2 was mostly detected in RIPA lysates and the medium samples at the highest concentration. It is critical to mention that a high molecular weight complex (>250 kDa) was usually detected (also seen in other studies) when probing medium samples with the annexin A2 antibody. Currently, we do not know the composition of this high molecular weight complex.

**Figure 3:**
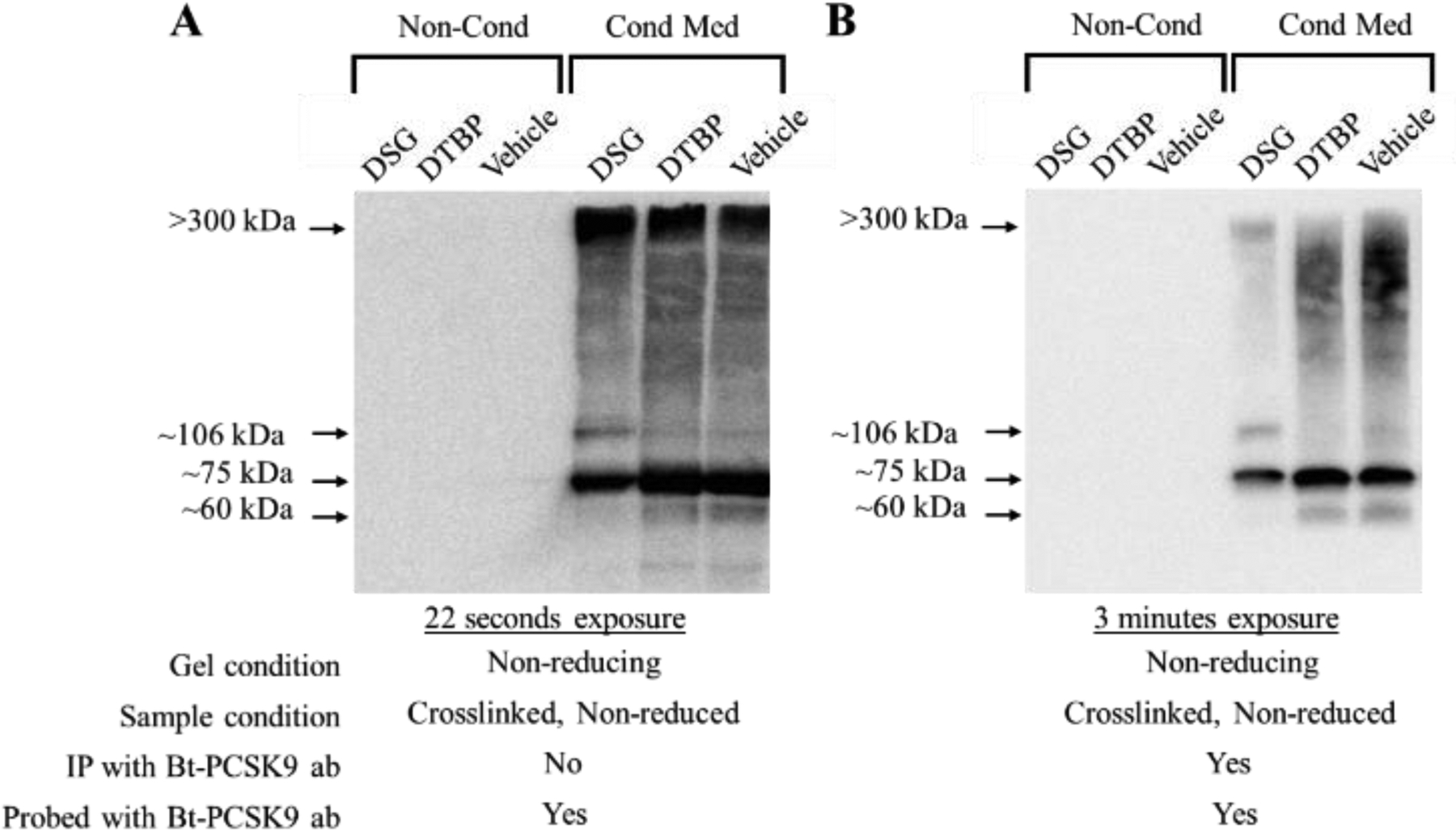
Crosslinking of PCSK9 to proteins secreted by C3A cells exposed to MITO+ medium. Two different crosslinkers, DSG and DTBP, were used. (A) A typical Western blot for PCSK9 is shown after cross-linking, neutralization of the crosslinker, and detection with a biotinylated (Bt) PCSK9 specific antibody. (B) A typical Western blot for PCSK9 is shown after cross-linking, neutralization of the crosslinker, immunoprecipitation (IP), and detection with a biotinylated (Bt) PCSK9 specific antibody. Both (A) and (B), sample preparation and electrophoresis were performed under non-reducing conditions. “Non-Cond” refers to samples prepared from an MITO+ medium that was not exposed to cells. The exposure time for each blot has been indicated.

**Figure 4:**
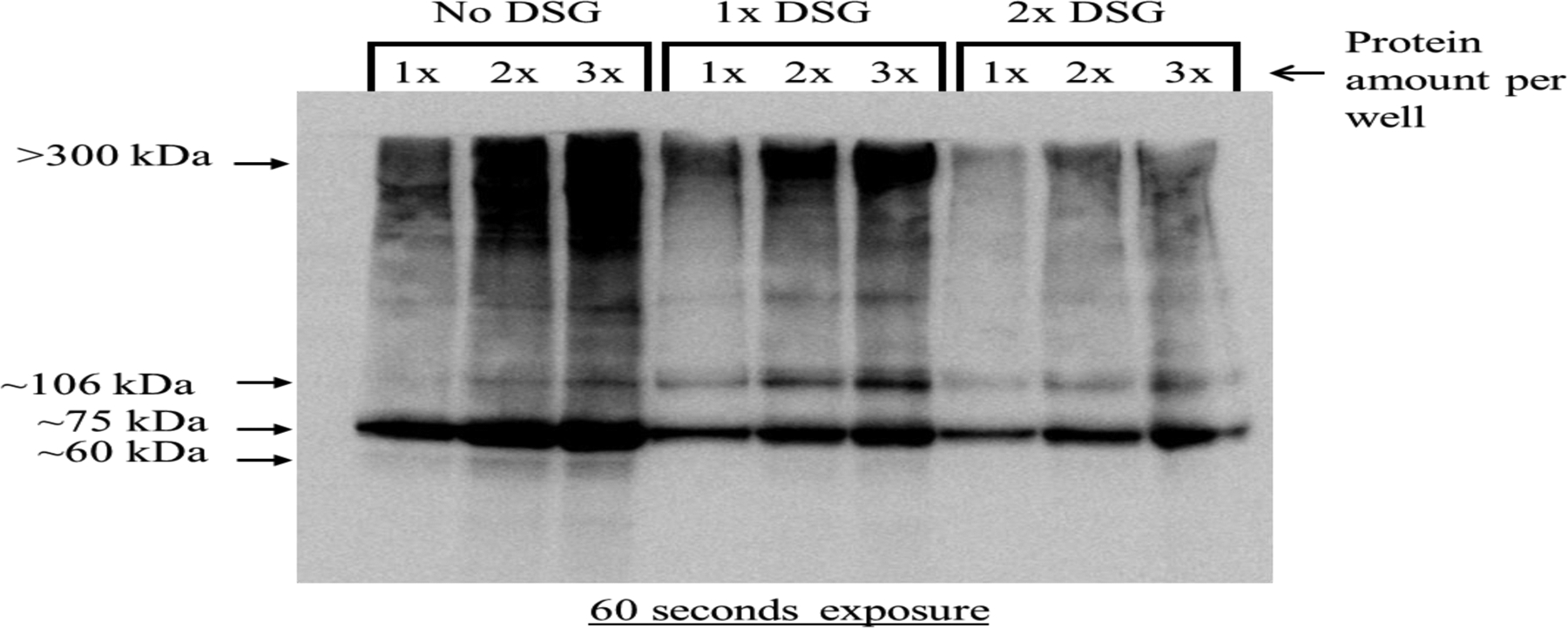
Crosslinking of PCSK9 to proteins secreted by C3A cells exposed to MITO+ medium. Increasing amounts of DSG (1 mM or 1x DSG and 2 mM or 2x DSG) were used. In this case, crosslinking reactions were carried out with increasing amounts of medium protein. A typical Western blot for PCSK9 is shown after cross-linking, neutralization of the crosslinker, and detection with a biotinylated (Bt) PCSK9 specific antibody. Sample preparation and electrophoresis were performed under non-reducing conditions. The exposure time for the blot has been indicated.

Next, we carried out cross-linking studies. For this, concentrated protein samples were mixed with two different crosslinkers, DTBP or DSG. After stopping the crosslinking reaction, aliquots of the crosslinked proteins were immunoprecipitated (IP) using biotinylated sheep anti-PCSK9 antibody. Crosslinked proteins, before and after IP, were electrophoresed using non-reducing conditions. Protein complexes with PCSK9 were analyzed using standard Western blotting analysis. Figure 3 illustrates that only DSG was successful in forming complexes between secreted hepatic proteins and PCSK9. Two major complexes (at about 106 kDa and >300 kDa, by comparing to protein markers) were seen both before and after IP. The cross-linking experiment was repeated, but this time, increasing amounts of DSG crosslinker (0, 1, and 2 mM) and medium proteins were used. As shown in Figure 4, once again, a complex at about 106 kDa, as determined by comparing to the protein marker, was observed indicating that PCSK9 was interacting with another protein found in the medium of the cells.

**Figure 5:**
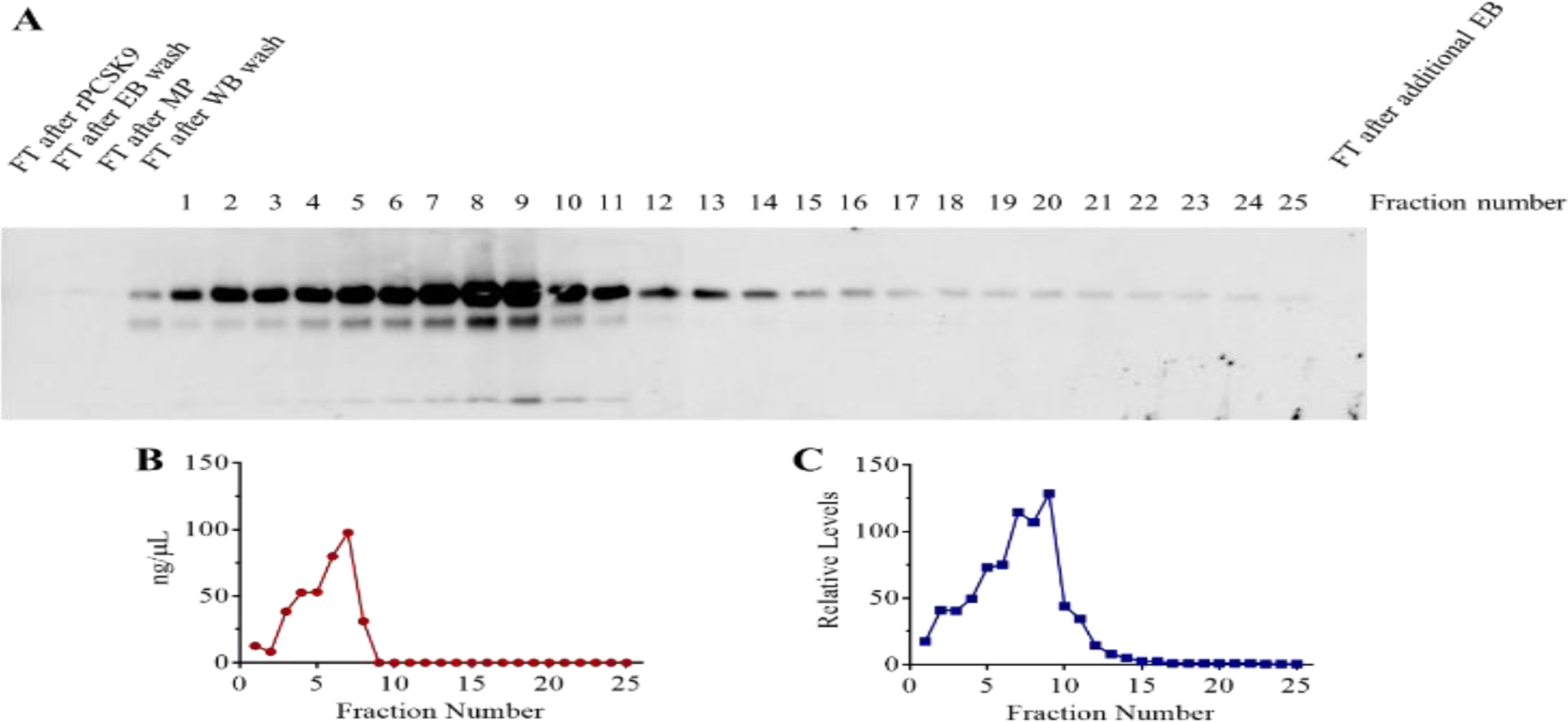
Affinity chromatography to isolate proteins secreted by C3A cells that interact with PCSK9. Twenty-five fractions were collected. (A) A typical Western blot for PCSK9 is shown. The primary antibody used in the Western blotting was mouse anti-PCSK9. “FT” stands for flow-through. “EB” stands for elution buffer. “MP” stands for medium protein. “WB” stands for washing buffer. (B) Total protein levels (ng/μL) determined using the BCA assay. (C) Relative PCSK9 levels (relative to the background signal) determined by quantitating the blot from (A).

The following step was to perform affinity chromatography to purify the protein(s) that interact with PCSK9. The entire process was carried as described in Materials and Methods. Electrophoresis and standard Western blotting analysis using the mouse PCSK9 antibody was performed, and the results are shown in Figure 5.

**Figure 6:**
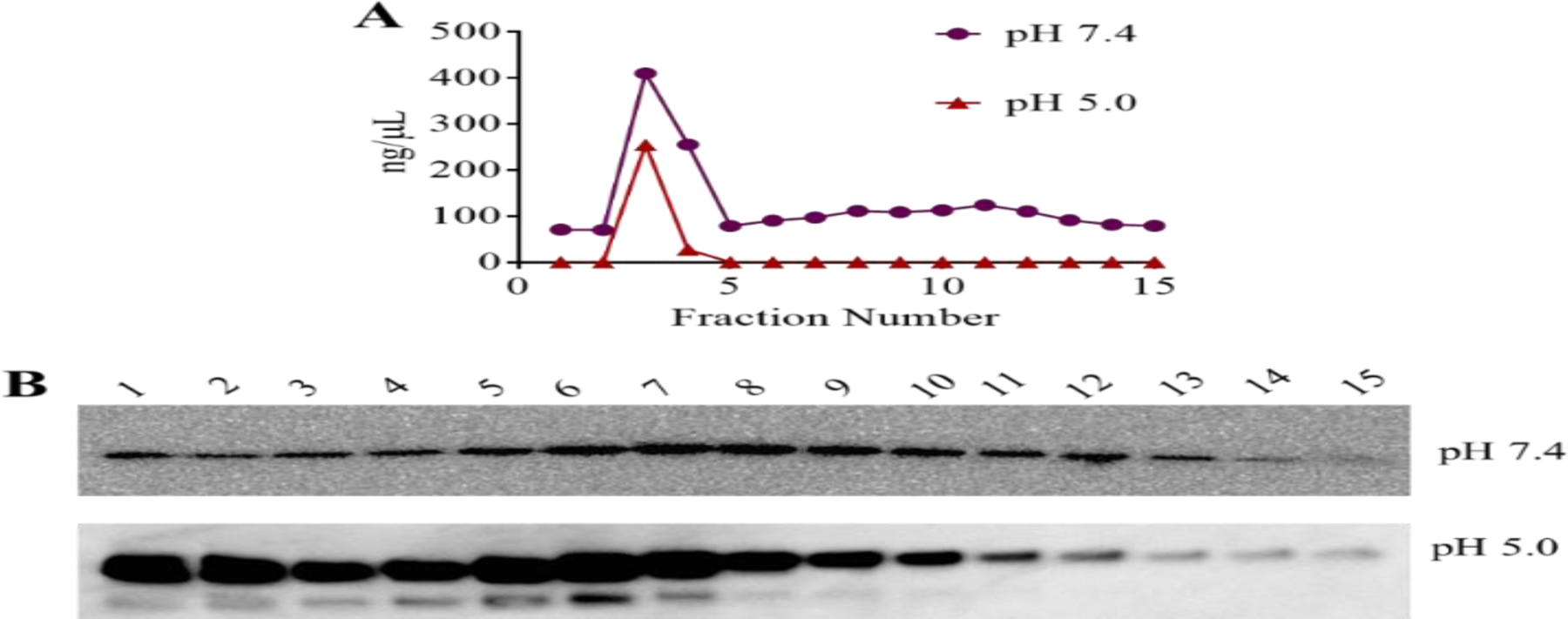
Affinity chromatography to isolate proteins secreted by C3A cells that interact with PCSK9 at different pH’s (pH 7.4 and pH 5.0). Twenty-five fractions were collected, but only 15 were analyzed. (A) Total protein levels (ng/μL) determined using the BCA assay. (B) A typical Western blot for PCSK9 is shown. The primary antibody used in the Western blotting was mouse anti-PCSK9.

As illustrated, fractions 3–8 had the highest levels of detectable proteins by the BCA assay. Fractions 2–12 had the highest levels of detectable PCSK9 protein by the Western blotting analysis. Affinity chromatography was also performed in the presence of concentrated serum samples with two different pHs (7.4 and 5.0). In this experiment, only the first 15 fractions were analyzed further. As illustrated in Figure 6A, the lowest amount of total protein binding to the column was seen in a column run under pH 5.0 as demonstrated by the BCA assay. However, the highest binding of the PCSK9 protein to the column was at pH 5.0 as evidenced using Western blotting analysis (Figure 6B).

Fractions 2–8 for each sample (pH 7.4 or pH 5.0) were mixed and concentrated using 3 kDa concentrators. After confirmation of the presence of PCSK9 in these samples (data not shown), they were sent out for protein identification using mass spectrometry [performed by DHMRI, Kannapolis, NC]. Several proteins were identified (Supplemental Data 1 & 2). From these proteins, four proteins were selected for further analysis (Table 1). These proteins were alpha-1-antitrypsin (A1AT), alpha-1-microglobulin/bikunin precursor (AMBP), selenoprotein P (SEPP), and apolipoprotein H (APOH). A1AT and AMBP were detected in both samples (pH 7.4 and pH 5.0). SEPP was only detected in the sample with pH 7.4, whereas APOH was detected in the sample with pH 5.0. These proteins are known to be synthesized and secreted by hepatic cells [27–30].

**Figure 7:**
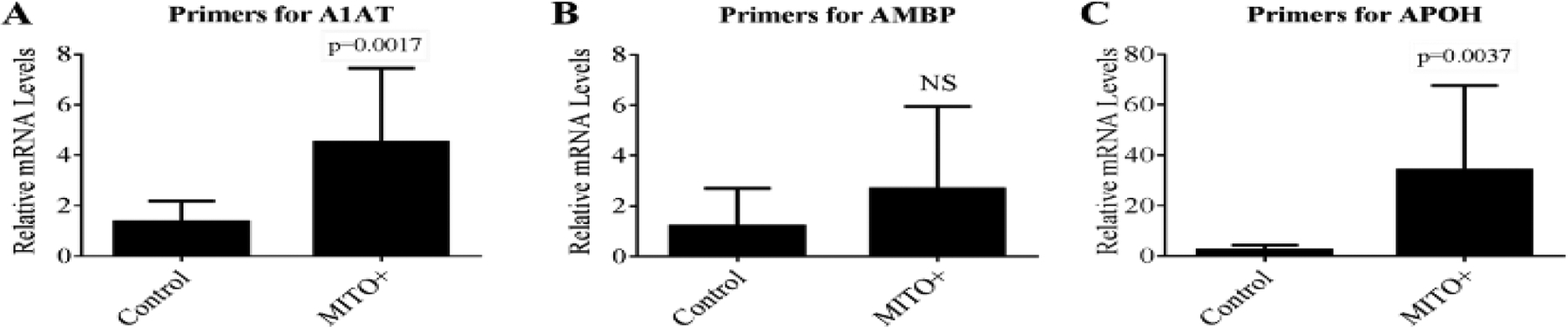
Quantitative real-time PCR results using primers for A1AT, AMBP, and APOH in C3A cells treated with Regular (control) and MITO+ media. RNA and ssDNA preparation and analysis using qRT-PCR were done using the methods described in the text. Calculations were performed employing the comparative Ct method and the data obtained with primers specific for 18s rRNA. Representative data are shown for n=12 per condition in the three graphs. This experiment was repeated four times.

Using the sequence information for the four genes encoding the identified proteins, PCR primers were designed and synthesized. RNA preparation from cells treated with Regular (control) and MITO+ medium, synthesis of ssDNA from the RNA samples, and qRT-PCR reactions were carried out using the methods described above. Amplification with primers specific for 18s rRNA was used for calculations employing the comparative Ct method. MITO+ treatment increased the expression of the A1AT mRNA by 3.3-fold (p=0.0017) and of the APOH mRNA by 14.3-fold (p=0.0037) (Figure 7A and 7C). MITO+ treatment caused a nonsignificant increase in AMBP mRNA levels (Figure 7B). We were unable to detect mRNA expression for SEPP even after using three different set of primers (data not shown). Thus, the follow-up studies concentrated on A1AT, APOH, and AMBP.

**Figure 8:**
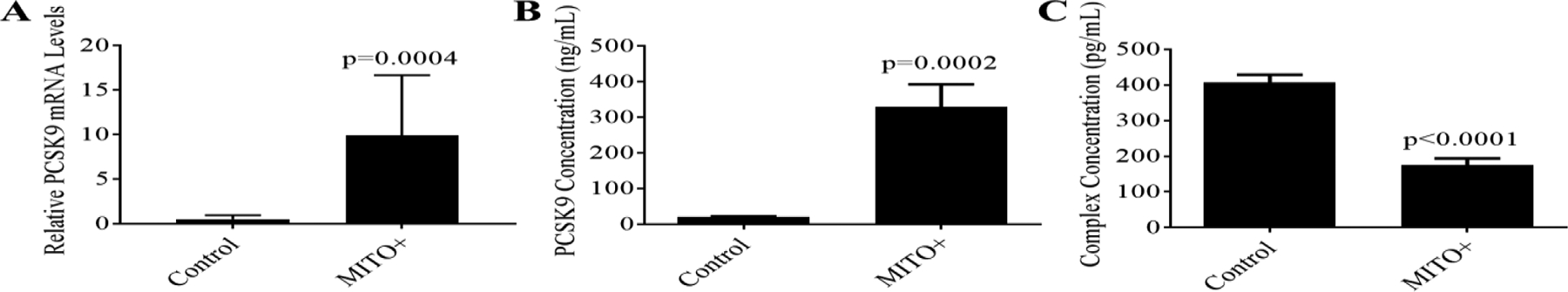
Expression and function of PCSK9 in C3A cells treated with Regular (control) and MITO+ media. (A) RNA and ssDNA preparation, analysis using qRT-PCR, and calculations were done using the methods described in Fig. 7. Representative data are shown for n=10 per condition. (B) Secreted PCSK9 levels in the media as determined using PCSK9 ELISA. (C) Complexes formed between PCSK9 and the LDL receptor in C3A cells as determined using Complex ELISA. Representative data are shown for n=9 per condition in (B) and (C). These experiments were repeated at least four times.

To confirm that the cells were responding to the treatments as previously reported [21], measurements of PCSK9 expression (mRNA and secreted levels) and activity (complex formation with the LDL receptor) were done. As expected, MITO+ treatment resulted in increases in PCSK9 mRNA levels by 19-fold (p=0.0004; Figure 8A) and in secreted PCSK9 levels by 16.2-fold (p=0.0002; Figure 8B). As previously reported [21], the number of complexes formed between PCSK9 and the LDL receptor in C3A cells treated with MITO+ medium was reduced by 57% (p<0.0001; Figure 8C). The subsequent step was to determine the expression levels of A1AT, APOH, and AMBP proteins. A1AT and APOH proteins were measured using standard Western blotting analysis.

**Figure 9:**
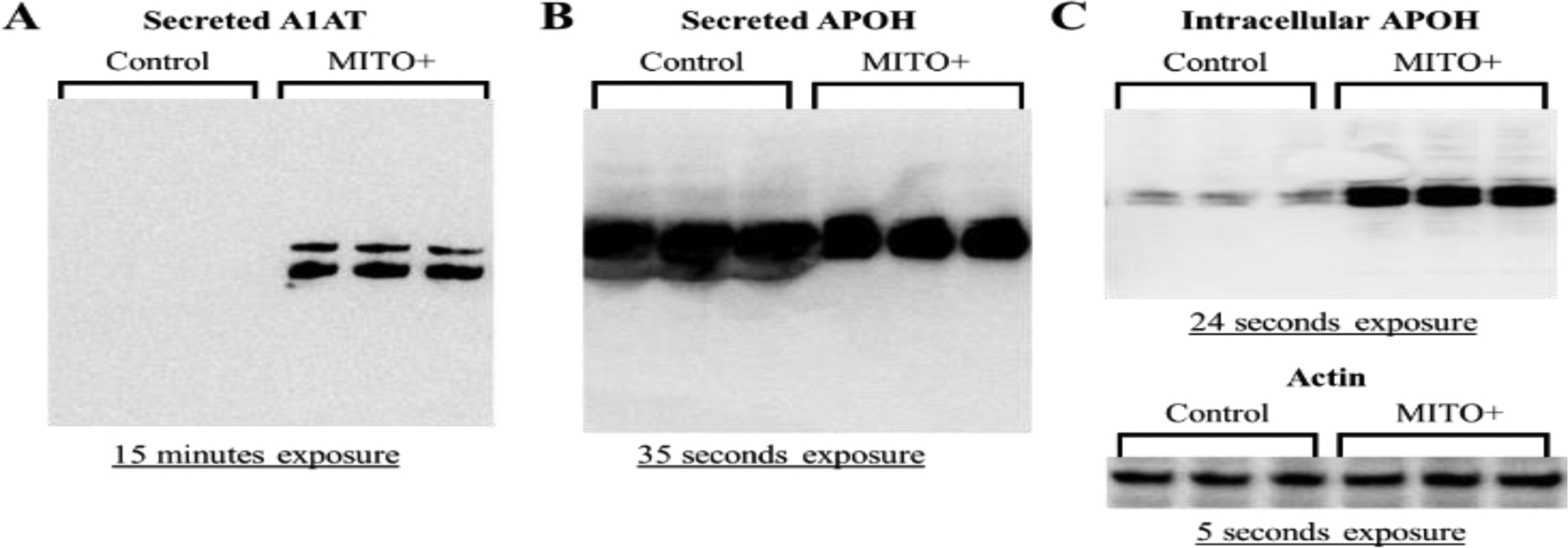
Expression levels of A1AT and APOH proteins in C3A cells treated with Regular (control) and MITO+ medium. Probing with the different antibodies was performed as described in Materials and Methods. (A) & (B) Expression levels in conditioned media samples. Conditioned media samples were precipitated using 50% ammonium sulfate and desalted using filtration. (C) Expression levels of APOH and the internal control actin in RIPA proteins. The exposure time for each blot has been indicated. These experiments were repeated three times.

As shown in Figure 9A and 9B, respectively, A1AT was only expressed in the conditioned medium of cells treated with MITO+, whereas APOH was expressed in all the samples. Interestingly, the amount of extracellular APOH protein was lower in the MITO+ than in the control cells (Figure 9B). Western blotting analysis of cellular lysates (RIPA proteins) indicated that the intracellular levels of APOH protein were higher in the MITO+ than control cells (Figure 9C) suggesting that under the MITO+ condition secretion of APOH was decreased, internalization of APOH was increased, or both.

**Figure 10:**
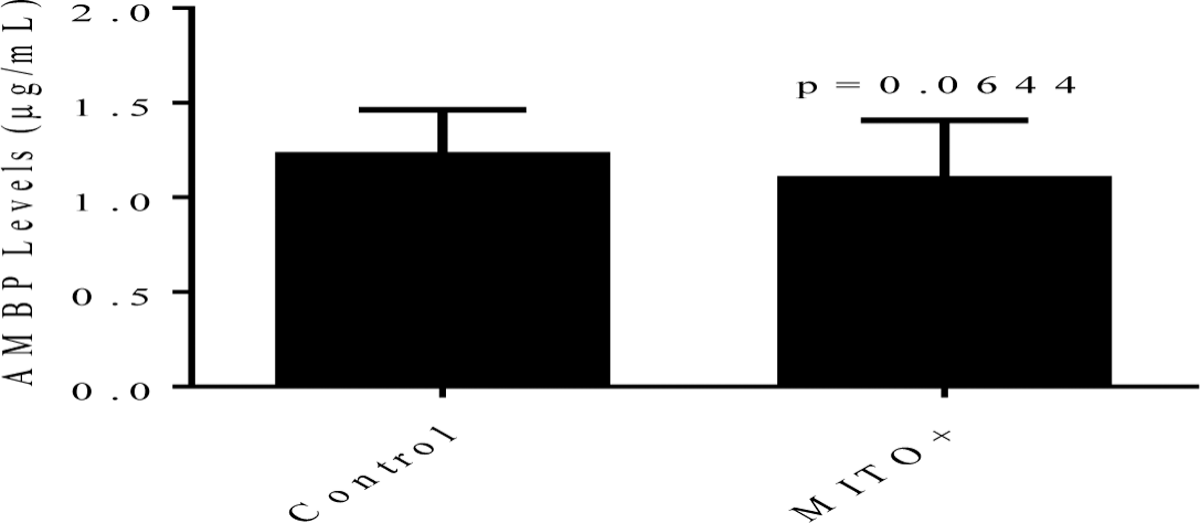
Secreted AMBP levels in conditioned media from C3A cells treated with Regular (control) and MITO+ medium as determined using AMBP ELISA. AMBP protein levels in diluted medium samples were determined as described under Materials and Methods. Representative data are shown for n=30 per condition.

The levels of AMBP protein in conditioned medium were measured using ELISA. In this case, the medium samples were not concentrated but diluted using reagent diluent provided by the kit. As shown in Figure 10, as for the mRNA, AMBP protein levels were not statistically affected (p=0.0644) by the treatments.

## Discussion

It has previously demonstrated that a factor(s) that is secreted from C3A cells upon treating with MITO+ medium inhibits the interaction between PCSK9 and the LDL receptor [21]. Herein, three proteins with the potential to function as the endogenous inhibitor(s) of PCSK9 were identified. These proteins were A1AT, APOH, and AMBP. The expression of A1AT and APOH, both at the mRNA and protein levels, was shown to increase in cells treated with the MITO+ medium as compared to treating with Regular (control) medium. However, AMBP expression did not change in response to the treatments. A1AT and AMBP were identified in samples interacting with PCSK9 at pH 7.4 and pH 5.0 suggesting that these proteins could interact with PCSK9 at the plasma membrane and within the endosome, respectively. The finding that APOH was identified in samples interacting with PCSK9 at pH 5.0 suggests that APOH may interact with PCSK9 mainly in the endosome. Additional studies are required to confirm the involvement of these proteins in PCSK9’s function.

A1AT is a protease inhibitor from the serpin superfamily [31,32]. As mentioned above, A1AT is synthesized and secreted primarily by the liver but also by neutrophils and monocyte/macrophages [31,32]. This serpin inhibits a wide variety of proteases [33] and protects tissues from inflammation by inhibiting enzymes such as neutrophil elastase [34]. Deficiency in A1AT is associated with chronic liver disease, cirrhosis, and hepatocellular carcinoma (HCC) in children and adults, and emphysema and chronic obstructive pulmonary disease (COPD) in adults [34,35]. These effects are mainly due to increased breakdown of elastin [34,35]. Recently, an association between A1AT levels and atherosclerosis started to catch interest. In fact, low A1AT levels have been associated with atherosclerosis development [36]. On the other hand, it has been shown that binding of A1AT to LDL can protect against atherosclerosis [37]. A1AT is internalized into target tissues via the scavenger receptor class B type 1 [38]. This inhibitor has a predicted molecular weight of 47 kDa, but another two forms of 40 and 35 kDa, respectively, produced as a result of alternative splicing, have been reported [39]. We have been able to see all three A1AT isoforms in Western blotting analysis (data not shown). Considering the expression pattern of A1AT in C3A cells in correlation with a decrease in PCSK9’s function, we proposed that A1AT could be the endogenous inhibitor of PCSK9 in our experimental system, but more experiments are required to determine this.

APOH, on the other hand, is a glycoprotein with a predicted molecular weight of 48 kDa. This is the size of the protein that we saw in Western blotting analysis. APOH is expressed and secreted to the plasma by the liver [30]. APOH appears to be mainly involved in coagulation, but whether it enhances or inhibits coagulation greatly depends on its conformation, which could be one of two different possibilities [40,41]. Also, antibodies against this glycoprotein have been detected in connection with infections like syphilis and autoimmune diseases as sclerosis, lupus, and antiphospholipid syndrome [42]. APOH levels increase with age and are found to be reduced during pregnancy and in patients that suffered a stroke or myocardial infarction [43]. This glycoprotein has been detected in atherosclerotic plaques and appears to accelerate atherosclerosis by binding oxidized LDL [44–46]. PCSK9 has similar roles in atherosclerosis development and increases with age as APOH does. Therefore, it would be interesting to determine the role of APOH on PCSK9 expression/function even though the levels of secreted APOH in C3A cells decreased instead of increased while the intracellular levels of APOH increased. Perhaps APOH operates as an inhibitor of PCSK9 before secretion of the convertase. However, at this point, we cannot reject APOH as a regulator of PCSK9.

The last protein identified, AMBP, is a very abundant serum glycoprotein, expressed and secreted mainly by hepatic cells [28]. This protein is considered a precursor protein that is cleaved into the alpha-1 microglobulin and bikunin [47]. Alpha-1 microglobulin has been associated with tissue protection against oxidative stress [48] and has a predicted molecular weight of 38 kDa. Bikunin has been linked to metastasis of tumor cells as well as coagulation [49]. No reports are linking AMBP and atherosclerosis. Although the data shown here suggested that AMBP may not be the endogenous inhibitor of PCSK9, more experimentation is required before discarding this protein as a regulator of PCSK9.

It is imperative to highlight that in addition to controlling the LDL receptor expression and serum LDL levels, PCSK9 appears to play conflicting roles in atherosclerosis development, inflammation, thrombosis, apoptosis, diabetes, obesity, hypertension, and Alzheimer’s disease (reviewed in [50]). PCSK9 also has positive functions including liver regeneration, protection against viral infections, and brain development [50–57], which could lead to serious side effect when this convertase is inhibited. Based on these functions, it is logical to assume that PCSK9 may interact with multiple proteins that could assist this convertase in mediating different roles, possibly, in a pathway-specific manner. The identification of these regulators of PCSK9’s function could lead to the development of new diagnostic tests to match patients with personalized treatment options while reducing side-effects. These regulatory proteins could also serve as the starting point in the design of more efficient drugs/pharmaceuticals against PCSK9 while allowing this convertase to perform its positive actions.

## Supplementary Material

Supplemental Data 1

Supplemental Data 2

## Figures and Tables

**Table 1:** Selected proteins identified by mass spectrometry.

Accession	−10IgP	Coverage (%)	#Peptides	#Unique	Average Mass (kDa)	Description
P01009 A1AT_Human (present in both samples)	49.31	14	4	4	46.8	Alpha-1-antitrypsin (SERPINAA1) PE=1 SV=3
P02760 AMBP_Human (present in both samples)	63.3	19	4	4	39	Alpha-1-microglobulin (AMBP) PE=1 SV=1
P49908 SEPP1_Human (present in the pH 7.4 sample)	21.47	6	1	1	43.2	Selenoprotein P (SEPP1) PE=1 SV=3
P02749 APOH_Human (present in the pH 5.0 sample)	26.95	4	1	1	38.3	Beta-2-glycoprotein 1 (APOH) PE=1 SV=3
